# Primary care quality improvement from a practice facilitator’s perspective

**DOI:** 10.1186/1471-2296-15-23

**Published:** 2014-02-03

**Authors:** Clare E Liddy, Valeriya Blazhko, Molly Dingwall, Jatinderpreet Singh, William E Hogg

**Affiliations:** 1 C.T. Lamont Primary Health Care Research Centre, Bruyère Research Institute, Ottawa, ON, Canada; 2 Department of Family Medicine, University of Ottawa, Ottawa, ON, Canada; 3 Faculty of Medicine, University of Toronto, Toronto, ON, Canada

**Keywords:** Practice facilitation, Practice facilitators, Cardiovascular health, Primary care, Quality improvement, Practice culture

## Abstract

**Background:**

Practice facilitation has proven to be effective at improving care delivery. Practice facilitators are healthcare professionals who work with and support other healthcare providers. To the best of our knowledge, very few studies have explored the perspective of facilitators. The objective of this study was to gain insight into the barriers that facilitators face during the facilitation process and to identify approaches used to overcome these barriers to help practices move towards positive change.

**Methods:**

We conducted semi-structured interviews with four practice facilitators who worked with 84 primary care practices in Eastern Ontario, Canada over a period of five years (2007–2012). The transcripts were analyzed independently by three members of the research team using an open coding technique. A qualitative data analysis using immersion/crystallization technique was applied to interpret the interview transcripts.

**Results:**

Common barriers identified by the facilitators included accessibility to the practice (e.g., difficulty scheduling meetings, short meetings), organizational behaviour (team organization, team conflicts, etc.), challenges with practice engagement (e.g., lack of interest, lack of trust), resistance to change, and competing priorities. To help practices move towards positive change the facilitators had to tailor their approach, integrate themselves, be persistent with practices, and exhibit flexibility.

**Conclusions:**

The consensus on redesigning and transforming primary care in North America and around the world is rapidly growing. Practice facilitation has been pivotal in materializing the transformation in the way primary care practices deliver care. This study provides an exclusive insight into facilitator approaches which will assist the design and implementation of small- and large-scale facilitation interventions.

## Background

The translation of evidence-based guidelines into clinical practice remains a significant challenge in primary care settings. Clinical guidelines often focus on a single disease and in some cases conflict with one another. Primary care providers often find guidelines difficult to interpret, particularly when caring for patients with multiple conditions. Even when a physician is aware of a new guideline, it can be difficult to make changes in a busy primary care practice. Practice facilitation has emerged as one method to bridge the gap between knowledge and practice [1].

Practice facilitation in health care is a quality improvement (QI) process that involves bringing an individual with expertise in change management and a solid understanding of the health care into a practice to assist the group in adapting their practices to optimize patient care delivery through increased adherence to evidence-based guidelines [2].

The origins of the practice facilitation model can be traced back to the Oxford Prevention of Heart Attack and Stroke project in England (1982–1984) [3,4]. Practice facilitators were described then as health care professionals who could help assess current processes and plan implementation measures to enhance prevention strategies and be cross pollinators of ideas and resource providers [3,4]. Practice Facilitators (PFs) now also known as Outreach Facilitators, Practice Enhancement Assistants, and Practice Coaches engage and build a partnership with providers and practices over time. They actively work with practices to help providers change their practice behavior to more readily and effectively adopt evidence-based approaches. The focus is on re-organization of practice for sustained delivery of quality care rather than increasing specific content knowledge and is often grounded in elements of the Chronic Care Model (CCM) [5] such as implementing planned care and recall, using a team approach, supporting patient self-management and integration with community resources.

Practice facilitation is gaining momentum worldwide, particularly across North America. Most of the Canadian provinces have implemented at least one facilitation program [6]. Similarly, the use of facilitation continues to grow across the United States, as numerous programs have been implemented by practice-based research networks (PBRNs), State health departments, professional associations, and health plans across the country. Furthermore, the United States Agency for Healthcare Research and Quality (AHRQ) has recently released a 'How-to’ guide on developing and running a facilitation program [2]. As the facilitation process is grounded in the establishment of a relationship with the practice, there is much that could be learned by exploring the perspectives of the practice facilitators themselves. There remain unanswered questions related to the potential reach of practice facilitation beyond the early adopters, the characteristics of the practices in terms of practice culture, readiness and other factors which could influence the uptake and effectiveness of facilitation.

In 2007, our team (principal investigators being Hogg and Liddy) implemented a practice facilitation trial called the Improved Delivery of Cardiovascular Care (IDOCC) through Outreach Facilitation. This was a primary care quality improvement initiative that aimed to assist providers in improving their delivery of cardiovascular care [7], specifically by focusing on increasing the adherence to evidence based care guidelines. A multi method evaluation was designed and included interviews with facilitators with the objective of gaining insight into their experiences of working with the practices.

The purpose of this qualitative paper is to report on the results of the facilitator interviews. We were interested in identifying the barriers the IDOCC facilitators faced during the facilitation process and subsequently the strategies that they used to overcome these barriers to help practices change. Our results will contribute to the knowledge base of this quality improvement approach and are relevant for those who are implementing practice facilitation programs in their regions.

## Methods

### The Improved Delivery of Cardiovascular Care (IDOCC) through Outreach Facilitation trial

The Improved Delivery of Cardiovascular Care (IDOCC) through Outreach Facilitation trial was designed as a stepped wedge cluster randomized control trial. It supported 84 diverse family practices with almost 200 primary care providers in improving their delivery of evidence-based cardiovascular care for patients at high risk, making this trial the largest facilitation study conducted in Canada. IDOCC used trained facilitators who worked with practices for 24 months to incorporate elements of the chronic care model into daily practice routines to improve the secondary preventive care for heart disease, stroke, peripheral vascular disease, chronic kidney disease, diabetes and for those at high risk for cardiovascular disease. Primary care practices were located throughout the Champlain region (Ottawa and its surrounding communities) of Ontario, Canada, a culturally diverse region with a population of 1.2 million people who have chronic disease burdens and patient health outcomes that are comparable to Ontario and the rest of Canada. Detailed information about the recruitment, participants and data collection can be found elsewhere [7].

In brief, all practices within the Champlain region were invited to participate in IDOCC through a postal or fax invitation. Practices were enrolled in the trial if at least one physician from the practice agreed to participate. In total, 194 physicians in 93 practices were enlisted to participate, with nine practices dropping out prior to the initiation of the study. Participating practices varied in practice team structure, physician remuneration approach, and rurality (Table 1).

**Table 1:** Breakdown of IDOCC practice characteristics

**Characteristic**	**n (%)**
EMR	41 (48.8%)
Practice structure	
Single physician	33 (39.3%)
Multi-Physician Group Practice	51 (60.7%)
Physician remuneration	
Fee-for Service	45 (52.4%)
Capitation	27 (32.9%)
Salary- Community Health Centres	12 (14.6%)
Urban practices	69 (82.1%)

### Timeline of study

The IDOCC study was conducted over several years due to the study design which had staggered start times so whilst the facilitators worked with each individual practice for a 24 month period, they worked as practice facilitators for the IDOCC project over five years (2007–2012).

### Qualitative data collection

All PFs who were involved in the IDOCC project were invited to participate in a face-to-face semi-structured interview. The interviews took place in Ottawa, Canada in the summer of 2012. The guide included mostly open-ended questions and was pre-tested and modified according to the feedback. The interview questions asked facilitators about their in-practice experiences of the intervention (see Additional file 1). Questions touched on areas such as common barriers faced, facilitation approaches that they used which resonated with practices, practice/provider level characteristics that they felt impacted practice engagement, motivational approaches, and effective modes of communication. Factors intrinsic to the practice such as electronic medical records (EMR) were also explored. All interviews were performed by one research assistant (MD) who was external to the original project team. The interviews were transcribed from the audio recording. The study was approved by the Ottawa Hospital Research Ethics Board.

### Analysis

A qualitative data analysis using the immersion/crystallization technique [8,9] was used to examine the interview transcripts. The transcripts were analyzed independently by three members of the research team. Immersion, the initial aspect of this technique, was conducted by reviewing the transcript for each interview, analyzing each interview from start to finish, and then moving on to the next transcript. The iterative analysis included cycles of reading, summarizing and rereading the data. Minor and major themes were identified as suggested by Pope & Mays et al. [10]. We open-coded relevant sections of each transcript indexing themes and categories emerging from the data. These sections of data were centred on certain facilitators’ behavior, phrases and examples that belonged to a more general phenomenon. Themes emerging from the data were noted along with supporting examples ensuring that one interview was not over-quoted. During the first meeting, main descriptive themes were discussed; the researchers agreed or disagreed on the various points brought up. We then returned to the transcripts for a second iteration of the analysis, again followed by a group discussion, which was followed by a third iteration of the analysis and final development of analytic themes [11]. Each time researchers met, meeting minutes were taken and distributed among researchers for future reference.

## Results

All four PFs who had taken part in the IDOCC project were interviewed. All were female with graduate level qualifications: Masters of Health Psychology; BA in Psychology and Masters in Nursing; Registered Nurse and Masters Degree (Religion and Culture); and Registered Nurse with Masters Degree. Three out of four facilitators did not have previous facilitation experience. There was a seven week training period for PFs based on a Canadian practice facilitation guide [12] with ongoing training and updates during the course of the IDOCC intervention. On average, each PF worked with 21 practices over a 12–24 month period, although not concurrently. All practices completed the intervention.

Common barriers identified by the PFs included organizational behaviour (team organization, team conflicts, etc.), practice accessibility (e.g., difficulty scheduling meetings, short meetings), challenges with practice engagement (e.g., lack of interest, lack of trust), resistance to change, and competing priorities (Table 2). PFs also discussed facilitating approaches that they used to overcome these common barriers, from which we identified four major strategies: tailoring, integration, persistence and flexibility (Table 2). Although we present these strategies separately for the purpose of clarity, in reality they are interdependent.

**Table 2:** Barriers and facilitating solutions

**Barrier**	**Facilitating solutions to overcome barriers**	Strategies*
**Organizational behaviour***(i.e., how team works together, is organized, hierarchy, conflicts, etc.)*	*In regards to dealing with conflict*: “Have an open discussion, have everybody voice their concern. If I wasn’t able to do it in the group then it would be 'Send me your challenges’ and then I said hereare some of the challenges that as a team we’re facing”	I
	*In regards to facilitating changes in large teams*: “For that kind of change, you would need the clinical lead… You see, individual people might sign up, but the head of that team might not. And you really need buy in at the highest level to do anything”	I
**Practice accessibility***(i.e., challenges scheduling meetings, not enough time to make significant change)*	“The main thing to really be effective is to not put it on their plate, you know, 'call me when you need something’. It’s more like 'is it okay for me to connect with you in two weeks?’”	P
	“Email worked really well too. Cause some things I did not really need to have a discussion”	F
	“And having the flexibility, so with one physician, I knew that Friday afternoon he was finished clinic at 12, so I would always make sure that I had that open…”	F
	“The other strategy in relation to the time piece is to really find out what the practices is working on already and how I can add to it”	I, T
	“And the administrative staff, I think it’s essential to get them on board, just even from the initial recruitment because then your work within the practice itself is so much easier”	I
**Practice engagement***(i.e., lack of interest, no buy-in, maintaining engagement throughout, lack of trust)*	“So if I already had a physician that was engaging… I know that I would ask them to put the word out, knowing that if it was peer-to-peer, it would always have more weight”	I
	“The more frequent contact I had with practices, it seemed the better they did. If there was a length of time where I didn’t see them…some of the changes would take a bit of time or wouldn’t happen” (OF1, Line 42)	P
	“If someone was very sort of data oriented and you didn’t come with this, then you would get a lot of resistance… if your approach to that practice doesn’t align with their practice culture, I think it would be harder to make progress”	T
	“The way that I tried to address these is review the program with them, state the goal of the program, how they could benefit from the goals and the work that would have to go into it… that did work for some of them”	P
	“It’s the analogy of a terrier. And I think that’s what you are, you just have to be - or like a sheepdog - you just have to keep going back and going back and going back”	P
	I think I didn’t put forth that kind of, “This is what you need to do” kind of thing, it was more “I’m here to help. I’m not here form the Ministry, I’m not a Pharma rep, I’m here to be able to provide support for some of the changes that you think you would like to change” and so by always framing it that way, I didn’t really get a lot of resistance”	I
**Resistance to change**	“Instead of me pushing through and saying 'no, I think it’s really important that you do that’ – it’s not about me, it’s about having the practice work on what they need”	F, T
**Competing priorities***(i.e., urgent priority arises that shifts practices focus, practices losing momentum)*	*In regards to the competing demands of the H1N1 outbreak* - “So although it wasn’t within our cardiovascular component, we actually provided practices with that information” (regarding H1N1) “It’s in that building a relationship that yes, we recognize and realize that something else has taken over and that we can’t really do anything about it but we can still be helpful”	F, I
	“I think to a large extent, you have to wait… very often, you can’t move forward until these other issues have resolved in some fashion, and you have to respect that”	F
	“I used summaries, especially between the first year, the intensive, and the sustainability. This is what you planned, and this is where you are. What of these five things you said you’re going to do have actually been done? And then present that back to the team. Going back to the data, to keep going back to the data to see where people started and have they achieved what they said they were going to achieve?”	P

*F = Flexibility, I = Integration, P = Persistence, T = Tailoring.

The first and most expansive strategy addresses tailoring as a solution to deal with barriers such as dealing with organizational behaviour, difficulties in engaging the practice, and overcoming resistance to change.

The way the practice behaved as a whole was one of the most prominent issues facilitators had to deal with. Facilitators had little control over the way the practice was organized including its teamwork, practice’s readiness, and leadership structure. To overcome some of these systematic challenges, PFs had to recognize that each practice was unique and had to tailor approaches and tools accordingly: “one size does not always fit all” [PF2-15]. Recognition of the importance of tailoring was important in helping engage practices and overcoming resistance to change: “If someone was very sort of data oriented and you didn’t come with this, then you would get a lot of resistance… if your approach to that practice doesn’t align with their practice culture, I think it would be harder to make progress” [PF4-88].

Integration into the practice team and office routines along with trying to build strong relationships with practice staff was helpful in addressing barriers related to practice accessibility and team conflicts. For example, all PFs mentioned that finding time to meet with providers was challenging, and better integrating into the team and their routines was often helpful: “… integrate something I was doing within already a set meeting time then that worked really well” [PF1-76]. PFs also noted the importance of building strong relationships with practice staff, particularly the administrative staff: “And the administrative staff, I think it’s essential to get them on board, just even from the initial recruitment because then your work within the practice itself is so much easier” [PF4-310]. Integrating meant ensuring that practice members felt that PFs were a part of their team; for PFs, this close relationship meant a better understanding of practice culture and identifying champions.

Champions tended to be recognized by their enthusiasm and volunteering to provide support in implementing positive change; they were often interested in the holistic functioning of the practice and patient outcomes. Facilitators all thought that generally, any practice member could be an effective champion: “it wasn’t always someone who had to be the highest up, in a position of defined leadership that could affect the change” [PF3-624]. Champions were looked upon to take an active role in implementing change but in also providing support in engaging others: “So if I already had a physician that was engaging… I know that I would ask them to put the word out, knowing that if it was peer-to-peer, it would always have more weight” [PF1-627].

Remaining persistent throughout the intervention was also noted as being important in dealing with a number of the barriers identified, including practice accessibility and practice engagement. PFs stressed the importance of being persistent when setting up meetings with practices, as this was important in maintaining ongoing contact: “The main thing to really be effective is to not put it on their plate, you know, 'call me when you need something’. It’s more like 'is it okay for me to connect with you in two weeks?” [PF1-108]. This persistence was important in helping practices achieve their goals: “The more frequent contact I had with practices, it seemed the better they did. If there was a length of time where I didn’t see them… some of the changes would take a bit of time or wouldn’t happen” [PF1-2]. Furthermore, PFs also highlighted the importance of remaining persistent in cases where practices were not engaged, as it often took time for some practices to embrace change: “You have to be like a terrier or a sheepdog - you just have to keep going back and going back and going back” [PF3-205].

Flexibility was an essential characteristic PFs demonstrated to be effective in scheduling meetings, dealing with those who resisted change, and handling unexpected competing priorities. Scheduling meetings and maintaining regular contact with busy practices required a great deal of flexibility: “And having the flexibility, so with one physician, I knew that Friday afternoon he was finished clinic at 12, so I would always make sure that I had that open” [PF1-90]. Remaining flexible also played an important role in working cooperatively with providers. PFs stressed the importance of not dictating what changes needed to be made, but instead, allowing the practice to work on areas that they felt were important: “Instead of me pushing through and saying 'no, I think it’s really important that you do that’ – it’s not about me, it’s about having the practice work on what they need” [PF1-247].

During the intervention, several unexpected events occurred that shifted the focus of the practices taking part in IDOCC, including the H1N1 influenza outbreak. During these unforeseen events, the PFs stressed the importance of remaining flexible: “I think to a large extent, you have to wait. Very often, you can’t move forward until these other issues have resolved in some fashion, and you have to respect that” [PF4-544]. In some cases, PFs adapted to the situation and provided support to practices in areas that were not a specific focus of the IDOCC project: “So although it wasn’t within our cardiovascular component, we actually provided practices with that information” (regarding H1N1) “It’s in that building a relationship that yes, we recognize and realize that something else has taken over and that we can’t really do anything about it but we can still be helpful” [PF1-186].

## Discussion

The results from this research provide insight into facilitation approaches from the perspective of the practice facilitators themselves. Common barriers encountered by PFs have been outlined in this study along with suggestions on how to get around these barriers to continue on the road to better patient care. Our findings suggest that tailoring, integration, persistence and flexibility are important strategies in overcoming common barriers faced by PFs.

Our study is not the first one to explore barriers and facilitators to practice change. Nevertheless, to the best of our knowledge, it is one of the first studies to solely focus on qualitative exploration of practice facilitators’ perspectives who worked with such a large number of primary care practices. We found a number of studies that looked at the role of PFs, the methods [13-16] used during facilitation, and the skills and attributes [17-19] facilitators need to have in order to substantially improve practice performance. Most of these studies did not use interviews with PFs as their data source. A study by Petrova et al. [20] looked at facilitation characteristics in palliative care that spoke to flexibility and tailoring approaches, but mainly focused on how different facilitation characteristics relate to the degree of change achieved by practices. A literature review by Wensing et al. [16] identified that the common strategies of feedback, reminders, and group education for improving the care provided by general practitioners, which were consistent with the results of our study. A review by Harvey et al. [18] identified many attributes including interpersonal and communication skills that are believed to be prerequisites for a PF. The importance of these skills for facilitation was also recognized in our study.

The results of this study support strong evidence for tailoring [13] when implementing a facilitation program within the practice. Kaissi at al. [21] suggests that quality improvement programs should be developed according to the type of practice culture. An important aspect in achieving positive change starts with facilitator’s understanding of practice culture. This does not imply that it is the responsibility of the facilitator to do a cultural intervention [22] but to adjust to what the practice is ready to take on depending on the way it functions. Understanding the organisational behaviour of the practice will help to set the stage for goal-setting and whether large or small projects should be taken on.

### Limitations

Several limitations should be taken into consideration when analyzing the results of our study. Our data is based on a data set, within one study thus limiting the generalizability of the results; however this is mitigated by the study design, size of the region and also the diversity of the included practices. Inability to determine the truthfulness and accuracy of interviewee’s responses is another limitation generally seen in these types of studies. Therefore, errors in self-report may have biased our findings. There is also potential for recall bias, even though three quarters of the facilitators were interviewed immediately after the intervention was complete.

## Conclusion

This study summarizes common barriers faced by PFs who took part in a large facilitation project conducted in Canada, and provides approaches used to overcome these obstacles. Common strategies identified in this study pointed to the importance of tailoring, integration, persistence and flexibility in overcoming common barriers faced in primary care practices. To the best of our knowledge, this is one of very few studies to comprehensively interview PFs to identify practical tips for engaging practices and overcoming common barriers. With the rapid growth of facilitation interventions across North America and worldwide, the pragmatic approaches outlined by the four experienced PFs in this paper will be a valuable guide for policy makers, program developers, and current and future PFs. Future evaluations of practice facilitation should continue to include qualitative aspects to increase our understanding of this approach.

## Competing interests

The authors declare that they have no competing interests.

## Authors’ contributions

CL and JS originally conceived of the study. WH and CL are the PIs on the IDOCC study. MD conducted the interviews and participated in drafting the paper. VB assisted with data collection, and drafting of the paper. The analysis was done by CL, JS and VB with CL overseeing the analysis process. All authors critically reviewed and approved the final manuscript.

## Pre-publication history

The pre-publication history for this paper can be accessed here:

http://www.biomedcentral.com/1471-2296/15/23/prepub

## Supplementary Material

Additional file 1 Interview Guide. Click here for file
