# Supplementary material for: Primary care quality improvement from a practice facilitator’s perspective
Source: BMC Fam Pract. 2014 Feb 3;15:23. doi: 10.1186/1471-2296-15-23 (PMC3931473; doi:10.1186/1471-2296-15-23)
Supplement: Additional file 1 — Interview Guide. [file 1471-2296-15-23-S1.docx]

**Additional file 1:** Interview Guide

**1.** Could you tell me about the barriers you faced as an outreach [practice] facilitator in helping the practices achieve positive change? Please answer the following questions for each identified barrier:

1. Were you able to overcome the barrier and what strategies did you use?
2. Which barriers were you unable to overcome?
3. Why do you think you were unable to overcome the barrier at that time? What did you try that didn’t work? How did you alter your approach or strategy to make efforts more effective?
4. What do you think would have assisted you in successfully overcoming this barrier? Was there anything else the IDOCC team could have done to help support you and the practice in overcoming this barrier?

**2.** Were there any factors intrinsic to the practice (e.g. eagerness to change/learn, helpful administrative staff, etc.) that helped facilitate positive change?

a) If so, how can they be used effectively during the facilitation process?

**3.** Were there any barriers that were especially prominent when working with certain practice models?

**4.** Did you feel that the presence or absence of an EMR system acted as a barrier?

a) If so, what approaches did you use to overcome this?

**5.** What challenges, if any, did you face when taking over a practice from another facilitator part way through the intervention?

**6.** Over time, did you notice any changes or trends in the barriers you encountered or the strategies you used to overcome them?

**7.** Is there anything else that we have not discussed that you would like to talk about?

**8.** Had you had any previous facilitation experience prior to your training and work on the IDOCC project?
